# FOXP3 and GARP (LRRC32): the master and its minion

**DOI:** 10.1186/1745-6150-5-8

**Published:** 2010-02-05

**Authors:** Michael Probst-Kepper, Jan Buer

**Affiliations:** 1 Institute for Microbiology, Immunology and Hygiene, Städtisches Klinikum Braunschweig gGmbH, Braunschweig, Germany; 2 Institute for Medical Microbiology, University Hospital Essen, Essen, Germany

## Abstract

The transcription factor FOXP3 is essential for the development and function of CD4^+^CD25^hi^FOXP3^+ ^regulatory T (T_reg_) cells, but also expressed in activated human helper T cells without acquisition of a regulatory phenotype. This comment focuses on glycoprotein-A repetitions predominant (GARP or LRRC32) recently identified as specific marker of activated human T_reg _cells, which may provide the missing link toward a better molecular definition of the regulatory phenotype.

Reviewers: Dr Jim Di Danto, Dr Benedita Rocha and Dr Werner Solbach.

## Introduction

### Proposed role of FOXP3 in self-tolerance

The maintenance of self-tolerance involves central and peripheral mechanisms, but the process of thymic negative selection is imperfect. Self-reacting clones that escape central tolerance are kept in check by a variety of peripheral tolerance mechanisms, in which the dominant tolerance exerted by CD4^+^CD25^+ ^regulatory T (T_reg_) cells plays an important role. The development and function of T_reg _cells crucially depends on the forkhead/winged helix transcription factor FOXP3. This dependence is compellingly illustrated by the fact that loss of function of FOXP3 leads to the development of the fatal autoimmune lymphoproliferative disorder IPEX (immunodysregulation, polyendocrinopathy, and enteropathy, X-linked) syndrome [1,2]. Similarly, loss of function of FoxP3 in mice, either natural (*scurfy*) or recombinant, results in an analogous immune pathology due to a lack of T_reg _cells [3-5]. The dominant role of FOXP3 is further corroborated by the fact that the gain of function induced by ectopic expression in conventional CD4^+^CD25^- ^helper T (T_h_) cells leads to the acquisition of suppressor function and the induction of a partial regulatory phenotype in mice and humans [3,6-8].

Despite advances in our understanding of T_reg_-cell lineage commitment and function, gained mainly by the study of FoxP3 knock-out/knock-in mice [4,5,9-11], the dominant role of FOXP3 in the human system and its suitability as a *bona fide *marker of human T_reg _cells have been questioned [8,12-14].

### Why should T_reg _cells need more than FOXP3?

The reasoning that more than FOXP3 is necessary for fully explaining the regulatory phenotype in humans is supported by several observations. First, T-cell receptor (TCR) stimulation of CD4^+^CD25^-^FOXP3^- ^T cells leads to the induction of FOXP3 without interfering with the expression of effector cytokines such as interleukin-2 and interferon-γ [15-17]. Second, the expression of FOXP3 by conventional CD4^+^CD25^- ^T_h _cells and even by T_h _lines and clones does not necessarily indicate the acquisition of suppressor function [8,12,17-19]. Third, and more specifically, it has recently been found that demethylation of a conserved FOXP3 intronic region may be a better marker for suppressor function than the differential expression of FOXP3 at the mRNA or protein level in T_reg _and T_h _cell clones [20]. Fourth, ectopic expression of FOXP3 in human T_h _cells does not lead to the establishment of a stable regulatory phenotype [8,12,21]. Finally, although the enhancement of FOXP3 expression by transforming growth factor-*β*1 (TGF-*β*1) in activated human CD4^+^CD25^- ^T_h _cells generates so-called induced T_reg _(iT_reg_) cells, this phenotype is rapidly lost [19,21,22]. Altogether, these observations suggest that more than FOXP3 is necessary for fully explaining the regulatory phenotype.

A plausible explanation for the qualitative and quantitative differences in the expression of FOXP3 in human T_h _and T_reg _cells, with their mutually exclusive effector and regulatory functions, is the presence of a T_reg_-specific higher-order regulatory network [13,14,23]. The possibility that T_reg_-specific control mechanisms can maintain sustained high levels of FOXP3 and can control the regulatory function has been addressed only recently with the identification of the glycoprotein-A repetitions predominant (GARP or LRRC32) receptor [21,24-28].

### Identification and characterization of GARP as a safeguard of FOXP3

GARP was identified based on the analysis of gene expression profiling of T_reg _and T_h _cells following TCR stimulation, since TCR stimulation does lead to the induction of their mutually exclusive functions [21,24]. GARP is specifically induced in CD4^+^CD25^hi^FOXP3^hi ^T_reg _cells and thus is a T_reg_-specific activation marker [21,24-27]. Because the expression of GARP is up-regulated in FOXP3-transduced T_h _cells, GARP obviously depends on FOXP3, and this finding suggests a potential contribution to the regulatory phenotype [21,29].

The function of GARP has been elucidated by ectopic over-expression in human alloantigen-specific T_h _cells and down-regulation of GARP in human antigen-specific T_reg _cells [21,29]. Retroviral over-expression of GARP in T_h _cells, after some rounds of TCR stimulation, leads to an efficient and stable reprogramming/transdifferentiation of the established effector toward the regulatory program. This finding is associated with constitutive expression of FOXP3, the β-galactoside binding protein lectin, galactoside-binding, soluble, 3 (LGALS3) [8,30], and the cysteine-endoprotease legumain (LGMN) [8] and with an extended T_reg_-signature with suppression of effector cytokine production and acquisition of regulatory functions similar to those of T_reg _cells [21,29]. Thus, a FOXP3-regulating gene has been identified in the human system, and this finding suggests that the GARP signaling pathway may have direct therapeutic applications [28,31] similar to those that have been described for CD83 in the murine system [32].

**Figure 1:**
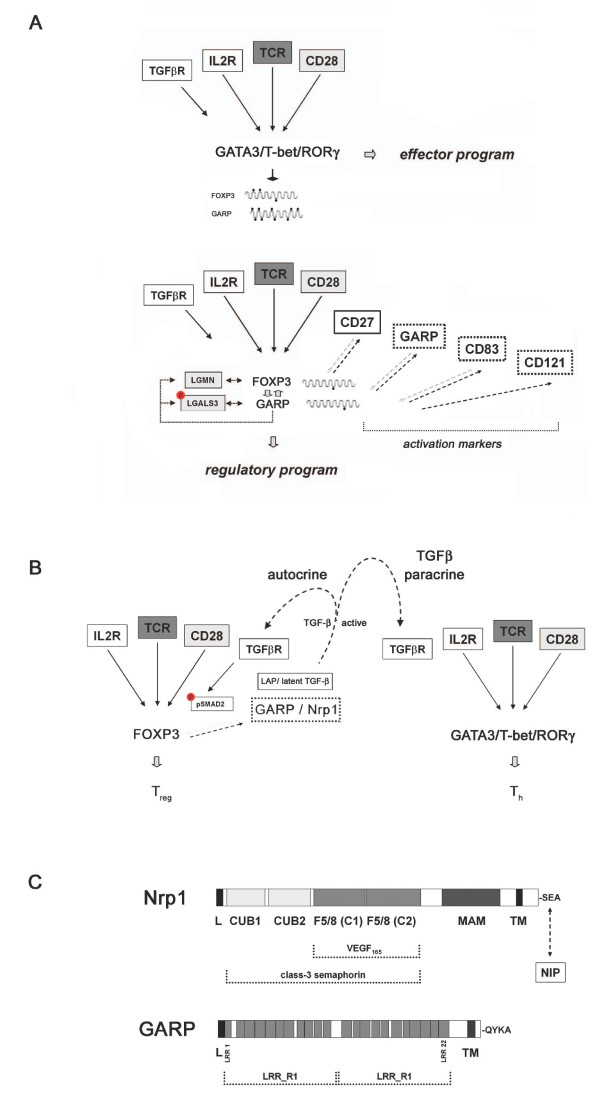
**GARP as safeguard of the regulatory phenotype**. (**A**) Qualitative (lineage-specific) and quantitative (dose-effect) differences in FOXP3 expression in human helper T cells (T_h_; upper panel) and regulatory T cells (T_reg_; lower panel) are due to lineage-specific methylation of the respective loci (indicated by black dots) and thus the expression of the genes FOXP3 and GARP. In human T_reg _cells, a positive feedback loop has been found between GARP and FOXP3; this feedback loop is interrelated with the FOXP3-enhancing molecules indicated. Phosphorylation of LGALS3 (indicated by a P) has been reported to be essential for the FOXP3-regulating function [21]. GARP is a receptor for LAP/latent TGF-*β *[26,27]. T-bet (T-box expressed in T cells), GATA3 (GATA-binding protein 3), RORγ (retinoic-related orphan receptor gamma) represent transcription factors of T_h_1, T_h_2, and T_h_17 cells. (**B**) Implications for human and murine T cells, respectively, of the autocrine and paracrine effects of GARP and Nrp1 surface-bound LAP/latent TGF-*β *after activation of TGF-*β*. The TGF-*β *signature phospho-SMAD2 (pSMAD2) has been observed in human (GARP^+^) and murine (Nrp1^+^) T_reg _cells. Paracrine effects include the generation of infectious tolerance. (**C**) Structural composition of the LAP/latent TGF-*β *binding proteins Nrp1 and GARP (LRR, leucine-rich repeat domain; CUB, complement subcomponents C1r/C1s domain; F5/8 coagulation factor domain; MAM, meprin, A5, and receptor protein-tyrosin phosphatase μ domain; TM, transmembrane region; L, leader peptide). NIP (Nrp1 interacting protein) is a Nrp1 binding protein involved in the regulation of Nrp1-mediated signaling as a molecular adapter [48].

In contrast, lentiviral down-regulation of GARP in human alloantigen-specific T_reg _cells by specific small interfering RNA (siRNA) substantially impairs suppressor function and FOXP3 expression that is associated with impaired induction of CD83 and CD27, both known to regulate FOXP3 [21,32,33]. More striking is the fact that similar changes are induced by the down-regulation of FOXP3 in T_reg _cells, a finding that provides compelling evidence for a GARP-FOXP3 positive feedback loop in T_reg _cells. Because this feedback loop seems to be interrelated with other FOXP3-regulating systems such as CD83 and CD27, the existence of a higher-order regulatory network as discussed recently by Hori [13], can be speculated (Figure 1a).

Thus, GARP is a specific marker of activated T_reg _cells and can explain the qualitative and quantitative differences in FOXP3 expression and function in T_reg _cells and T_h _cells. Lack of GARP expression further differentiates and explains the transient nature of TGF-*β*-induced iT_reg _cells [19], suggesting that natural and iT_reg _cells may represent alternative differentiation stages of suppressor cells [21,26]. In line with these observations, a specifically hypomethylated region in intron 1 of GARP and two differentially methylated regions with enhancer functions in T_reg _cells have recently been characterized. These findings have identified an epigenetic basis for the lineage-specific difference in GARP expression [34].

### GARP brings latent TGF-β into a new game

Members of the TGF-*β *family are pleiotropic cytokines with crucial functions in differentiation, morphogenesis, and immune homeostasis. Their function in immune homeostasis is evidenced by the fact that dysregulation of TGF-*β *functions is associated with autoimmunity [35]. Like many other cell types, human T_reg _and T_h _cells can secrete latent TGF-*β *[20,36]. However, latency-associated peptide (LAP) prevents the activation of latent TGF-*β *toward the active mature TGF-*β *[37]. The selective induction of LAP and the activation of active TGF-*β *upon TCR stimulation has been reported only for human T_reg _cells and clones [20,37,38]. This finding further explains the TGF-*β *specific transcriptional signature and detection of expression of phosphorylated SMAD2 in T_reg _cells of human and murine origin [20,23,37,39]. Moreover, specific up-regulation of LAP on activated human T_reg _cells has been shown to allow improvements in the purity of T_reg _cell isolation procedures by separating activated LAP^+^FOXP3^+ ^T_reg _cells from contaminating effector LAP^-^FOXP3^+/lo ^T_h _cells [38]. Thus, besides being a marker of activated T_reg _cells in complex with LAP, TGF-*β*1 is an important modulator of FOXP3 expression [19,21,40], and a potential mediator of infectious tolerance by its action in converting FoxP3^- ^murine CD4^+ ^T cells into functional FoxP3^+ ^iT_reg _cells [41].

The issue of cell-surface binding of LAP/latent TGF-*β *on human T_reg _cells has been addressed only recently with the identification of GARP as a receptor of this complex [26,27]. Therefore, two important regulatory circuits of T_reg _cells come together: the GARP-FOXP3 feedback loop and the autocrine TGF-*β *loop (Figure 1b). With that, the potential synergy of many of the T_reg _signature components that are essential for the regulatory properties that have been ascribed to TCR activation, interleukin-2, TGF-*β*, and FOXP3 itself [20,23] could be explained by this particular spatiotemporal interplay of interrelated signaling systems on T_reg _cells.

### Perspective: does Nrp1 represent a functional homologue of GARP in murine T_reg _cells?

The identification of GARP as a receptor for LAP/latent TGF-*β *opens speculation about a potential common denominator. The reason for such speculation is that neuropilin 1 (Nrp1), which has been characterized as a marker of CD4^+^CD25^+ ^T_reg _cells in mice that enhances T_reg _cell/dendritic cell contact during antigen recognition [42,43], has also been characterized as a receptor for LAP/latent TGF-*β *[39]. Moreover, murine sorted Nrp1^- ^T cells capture soluble Nrp1 (applied as a constant fragment [Fc]-fusion protein), and the captured Nrp1 increases their ability to bind LAP/TGF-*β*1. Such coated T cells acquire strong regulatory activity [39]. Thus, Nrp1 is a TGF-*β*1 receptor that, unlike GARP [27], also activates the latent form of TGF-*β*1.

The structural differences between GARP, a Toll-like receptor homologue with leucine-rich repeats, and the multi-domain protein Nrp1, a receptor for class-3 semaphorin-family proteins and vascular endothelial growth factor (Figure 1c) [44], necessitate further biochemical and molecular analyses to identify specific binding interactions and potentially associated molecules. The issue of GARP expression in murine T_reg _cells at the protein level remains to be elucidated.

## Discussions

### Open Questions Concerning the Function of GARP

Because GARP is constitutively expressed on platelets [26,45], and because a potential function of GARP on thrombus formation has recently been reported [46], important questions about GARP signaling and parallel function in platelets compared to T_reg _cells could be considered. Concerning the dependence of GARP expression on FOXP3 as described above, it has been reported that FOXP3 is expressed by human and murine megakaryocytes [47]. Therefore, the thrombocytopenia and platelet abnormalities experienced by some patients with IPEX syndrome can be explained by loss of function of FOXP3 [47]; however, this explanation would suggest a concomitant impairment of GARP expression and function on IPEX platelets, an impairment that has not yet been shown.

A controversial issue is the potential suppressor function of GARP, suggested recently [24], because platelets as a natural source of GARP-expressing cells do not function as suppressor cells [21]. Therefore, differences in the function of GARP in platelets and T_reg _cells can be suggested. This question is important for the potential design of GARP-selective drugs that can specifically target only T_reg _cells and not platelet functions. As of now, neither the ligand nor the signaling system or potential co-receptor(s) of GARP has been identified, and identifying them is the most important challenge for the future.

## Conclusions

The identification of GARP as a lineage-specific key receptor of human activated T_reg _cells, which is in part controlled by lineage-specific hypomethylation of the GARP locus, the characterization of a GARP-FOXP3 positive feedback loop that safeguards FOXP3 expression in T_reg _cells, and the binding of LAP/latent TGF-*β *to GARP provide a conceptual framework for a new molecular definition of the regulatory program. If GARP is a receptor for the well-known immune modulator TGF-*β *on human T_reg _cells, and if Nrp1 plays this same role on murine T_reg _cells, then this similarity might explain the similarities in the TGF-*β *signatures that have been observed in T_reg _cells in both species. The surface binding and activation of TGF-*β *have obvious implications for infectious tolerance. Complete elucidation of the GARP/GARP-ligand signaling system in T_reg _cells and platelets is an important challenge and a prerequisite for the future development of strategies and tools for inducing or inhibiting T_reg _cells in chronic infection, tumor immunotherapy, autoimmune diseases, and transplantation.

## List of abbreviations used

GARP: glycoprotein-A repetitions predominant; IPEX: immune dysregulation, polyendocrinopathy, enteropathy, X-linked; iT_reg _cells: induced regulatory T cells; LAP: latency-associated peptide; Nrp1: neuropilin 1; SMAD: Sma- and Mad-related protein 2; TCR: T-cell receptor; TGF-*β*: transforming growth factor-*β*; T_h _cells: helper T cells; T_reg _cells: regulatory T cells.

## Competing interests

The authors declare that they have no competing interests.

## Authors' contributions

MPK and JB both were involved in drafting of the manuscript and approved the final version to be published.

## Reviewers' comments

### Reviewer's report 1

Reviewer 1: Dr. Jim Di Santo, Cytokines and Lymphoid Development Lab, Institut Pasteur, Paris, France

Reviewer's comment: I have read your comment for Biology Direct entitled "FOXP3 and GARP (LRRC32): the master and its minion". I find this comment to be interesting and suitable for publication in Biology Direct. I do not have any specific comments for web publication.

### Reviewer's report 2

Reviewer 2: Dr. Benedita Rocha, Institut National de la Santé et de la Recherche Médicale (INSERM) U591, Insitut Necker, France

Reviewer's comment: Regulatory cells have a fundamental role in many disease processes, and extensive experimental and clinical data indicate that tools preventing or increasing regulatory function will have a fundamental therapeutic role. While FOXP3 expression generally correlates with regulation in the mouse, this is not so in human T cells. This comment reviews a fundamental aspect, the factors besides FOXP3 expression that ensure the induction of a stable regulatory function on human cells, their correlation with FOXP3 regulation and their possible mechanisms of action. I found the topic actual and important, the comment clear and comprehensive and strongly support publication.

### Reviewer's report 3

Reviewer 3: Dr. Werner Solbach, Insitute for Medical Microbiology and Hygiene, University Lübeck, Germany

Reviewer's comment: The ms. deals with the connectivity of FOXP3 and "glycoprotein - A repititions predominant receptor (GARP or LRRC32) in the context of the functioning of regulatory T cells (Tregs). This topic is of great relevance in the context of clarifying the equivocal role of FOXP3 and its partners for explanation of regulatory T cell circuits. It is clear that not only FOXP3 or GARP alone, but also latently activated TGF-*β *is crucial for the suppressive activity of Treg cells. The authors now very nicely bring together the GARP - FOXP3 regulatory circuit with the autocrine TGF-*β *loop which helps to explain many Treg features. In their perspective, they also try to open thoughtful avenues how to bring together (human) GARP with its possible functional homologue in mice, neuropilin 1. In summary, they present a valuable conceptual framework for understanding the regulatory program in the T cell reactivity system and beyond. The ms. is well written and easy to understand. I recommend publication in *Biology Direct*.
